# Breathless Strength: Ultrasonographic Insights into Expiratory Muscle Dysfunction in Spinal Cord Injury

**DOI:** 10.3390/medicina61050897

**Published:** 2025-05-15

**Authors:** Burak Kutuk, Kadriye Ones, Yunus Emre Dogan

**Affiliations:** 1 Department of Physical Medicine and Rehabilitation, Health Sciences University, Physical Therapy Rehabilitation Training and Research Hospital, 34903 Istanbul, Turkey; kadriyones@yahoo.com; 2 Department of Physical Medicine and Rehabilitation, Health Sciences University, Fatih Sultan Mehmet Training and Research Hospital, 34752 Istanbul, Turkey; ynsemredgn91@gmail.com

**Keywords:** spinal cord injury, ultrasonography, expiratory muscle strength, abdominal muscles, respiratory dysfunction, maximal expiratory pressure

## Abstract

*Background and Objectives:* This study aimed to evaluate the predictive value of ultrasonographic abdominal muscle thickness and thickening ratios for expiratory muscle strength in SCI patients. *Materials and Methods:* A case-controlled, cross-sectional study was conducted with 36 SCI patients and 30 age- and sex-matched healthy controls. Ultrasonographic measurements of the rectus abdominis (RA), external oblique (EO), internal oblique (IO), and transversus abdominis (TrA) were performed at rest and during forced expiration. Maximal inspiratory pressure (MIP) and maximal expiratory pressure (MEP) were measured to assess respiratory muscle strength. Correlation and regression analyses were performed to determine the relationship between ultrasonography (USG) parameters and expiratory muscle function. *Results:* SCI patients exhibited significantly lower MIP (76.27 ± 29 cmH_2_O vs. 91.63 ± 17.3 cmH_2_O, *p* = 0.007) and MEP (64.52 ± 21.55 cmH_2_O vs. 119.1 ± 26.48 cmH_2_O, *p* < 0.001) compared to healthy individuals. Ultrasonographic measurements revealed a significant reduction in forced thickness and thickening ratios of EO, IO, and TrA muscles in SCI patients (*p* < 0.001). MEP was positively correlated with EO forced thickness (r = 0.333, *p* = 0.047), IO forced thickness (r = 0.501, *p* = 0.002), and TrA forced thickness (r = 0.530, *p* = 0.001). Multiple linear regression analysis identified TrA forced thickness as the strongest predictor of MEP (β = 0.49, *p* = 0.001). *Conclusions:* Ultrasonographic measurements of abdominal muscle thickness and thickening ratios provide valuable insights into expiratory muscle dysfunction in SCI patients. TrA forced thickness demonstrated the strongest association with MEP, suggesting its potential as a novel, non-invasive biomarker for expiratory muscle weakness. These results support the use of USG as a practical clinical tool for guiding respiratory assessment and rehabilitation strategies in patients with spinal cord injury.

## 1. Introduction

Spinal cord injury (SCI) is a severe neurological disorder that results in significant motor, sensory, and autonomic dysfunctions, leading to long-term disability and reduced quality of life [1,2]. Depending on the level and severity of the injury, patients experience varying degrees of muscle paralysis, loss of voluntary movement, and compromised physiological functions, including respiration [2,3,4]. Respiratory complications are among the leading causes of morbidity and mortality in SCI patients, particularly those with injuries at or above the T12 level [5,6]. These complications arise due to impaired neural control over the respiratory muscles, which include both inspiratory and expiratory muscle groups.

The impact of SCI on respiratory function is multifaceted. Higher-level injuries, especially those affecting the cervical and upper thoracic regions, can severely impair the diaphragm and intercostal muscles, leading to reduced tidal volume and respiratory insufficiency [7,8]. Expiratory muscles, including the rectus abdominis (RA), external oblique (EO), internal oblique (IO), and transversus abdominis (TrA), play a critical role in forced expiration and the cough reflex, which is essential for clearing pulmonary secretions [9,10]. Weakness or paralysis of these muscles results in ineffective cough, mucus retention, and an increased risk of pneumonia, atelectasis, and respiratory failure [11,12].

Maximal inspiratory pressure (MIP) and maximal expiratory pressure (MEP) are widely used as indicators of respiratory muscle strength. Studies have shown that SCI patients exhibit significantly reduced MIP and MEP values compared to healthy individuals, which correlates with their reduced ability to generate effective airway clearance [13,14,15,16]. Although spirometry and electromyography (EMG) are commonly used to evaluate respiratory muscle function, they have limitations in terms of accessibility, cost, and the need for patient cooperation, particularly in those with severe disability [17,18].

Recent advances in medical imaging have highlighted ultrasonography (USG) as a non-invasive, accessible, and cost-effective tool for assessing muscle function. Ultrasonographic evaluation of the abdominal muscles provides real-time visualization of muscle thickness and contractile function, which can serve as an indirect measure of respiratory muscle strength [19,20]. Several studies have demonstrated the clinical utility of USG in assessing diaphragm dysfunction and inspiratory muscle impairment in SCI patients [17,21,22]. However, the predictive value of ultrasonographic abdominal muscle measurements in assessing expiratory muscle strength remains unclear.

Ultrasonographic measurement of abdominal muscle thickness and thickening ratio during respiration could offer a novel approach to evaluating expiratory muscle function in SCI patients [23]. The thickening ratio, calculated as the difference between forced end-expiratory thickness and resting thickness relative to resting thickness, has been proposed as a functional indicator of muscle activation [10]. Identifying a correlation between USG-measured abdominal muscle parameters and expiratory muscle strength (MIP/MEP) could provide clinicians with a simple and non-invasive method for respiratory assessment in SCI patients.

In this study, the aim was to evaluate the predictive value of ultrasonographic abdominal muscle thickness and thickening ratios in assessing expiratory muscle strength in SCI patients. By comparing these parameters with those of healthy controls, we aimed to determine whether ultrasonographic measurements can serve as a reliable, non-invasive tool for assessing respiratory dysfunction in this population.

## 2. Materials and Methods

### 2.1. Study Design and Study Population

This study was approved by the Bakirkoy Dr. Sadi Konuk Training and Research Hospital Clinical Research Ethics Committee (protocol number: 2021/524). All procedures involving human participants were conducted in accordance with the Declaration of Helsinki and its later amendments. Written informed consent was obtained from all participants prior to study enrollment.

This case-controlled, cross-sectional clinical study was conducted between 1 December 2021 and 1 December at the SCI Clinic of the University of Health Sciences Istanbul Physical Therapy and Rehabilitation Training and Research Hospital. A priori sample size calculation was performed using G*Power 3.1.9.7 software. To detect a large effect size (Cohen’s d = 0.8) with a power of 80% and a significance level of 0.05 in a two-tailed independent samples *t*-test, a minimum of 26 participants per group was required. The final sample size of 36 SCI patients and 30 healthy controls exceeded this threshold, ensuring adequate statistical power for the analyses.

Participants were included in the study if they were between 18 and 75 years of age, had a Mini-Mental Test Score of 24 or higher, and had an SCI level above T12. The exclusion criteria included a history of acute or chronic lung disease, thoracic or abdominal surgery, spinal shock, autonomic dysreflexia, or neuromuscular disease. Additionally, individuals with psychiatric disorders, such as depression or anxiety, that could influence cognitive function were excluded. The control group comprised healthy volunteers matched for age and sex with the SCI patients, with no history of neuromuscular or respiratory diseases and no previous thoracic or abdominal surgery.

A detailed evaluation form was completed for each participant, and demographic data, including age, gender, height, weight, and body mass index (BMI), were recorded. Ultrasonographic assessments were performed on the dominant side of the abdominal respiratory muscles. Respiratory muscle strength was evaluated by measuring maximal inspiratory pressure (MIP) and maximal expiratory pressure (MEP) in all participants.

### 2.2. Ultrasonographic Evaluation

**Figure 1:**
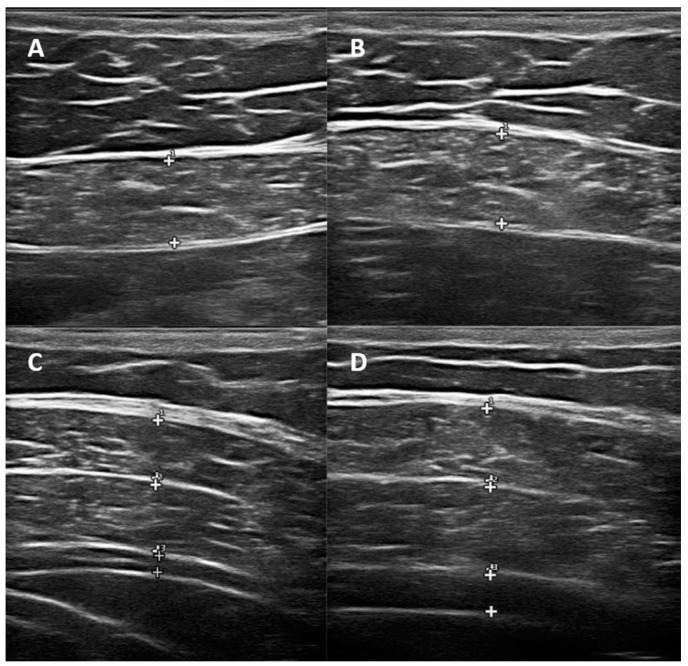
(**A**) Normal end-expiratory RA thickness. (**B**) Forced end-expiratory RA thickness. (**C**) Normal end-expiratory lateral abdominal muscle thickness. (**D**) Forced end-expiratory lateral abdominal muscle thickness. The (+) markers indicate the borders between anatomical layers used to measure muscle thickness. In panels A and B: **1**—Superficial fascia; **2**—Rectus abdominis muscle; **3**—Posterior fascia of the rectus abdominis. In panels C and D: **1**—External oblique; **2**—Internal oblique; **3**—Transversus abdominis.

Ultrasonographic measurements were performed using an Esaote MyLab70XVision ultrasound device with a 7–12 MHz linear probe. The participants were placed in a supine position, and muscle thickness was measured at the end of tidal expiration and forced expiration. The abdominal muscles assessed included the rectus abdominis (RA), external oblique (EO), internal oblique (IO), and transversus abdominis (TrA). For RA muscle thickness measurement, the ultrasound probe was placed 4 cm lateral to the umbilicus in the transverse plane. For EO, IO, and TrA measurements, the probe was positioned 2.5 cm anterior to the highest point of the iliac crest, centered on the midaxillary line. Measurements were obtained perpendicular to the hyperechogenic fascia lines, and all values were recorded in millimeters (mm) [24]. Each ultrasonographic measurement was performed three times by the same physician, who was blinded to group assignment, and the average value was used for statistical analysis to minimize observer bias. The thickening ratio (TR) was calculated as the difference between the forced end-expiratory thickness and resting thickness, divided by the resting thickness (Figure 1).

### 2.3. Respiratory Muscle Strength Assessment

Respiratory muscle strength was assessed using the MicroRPM Respiratory Pressure Meter (MicroDirect Inc., Lewiston, ME, USA), which measured MIP and MEP in cmH_2_O units. Each measurement was repeated three times, with the highest value recorded. The participants were instructed to perform the test with maximal effort, and verbal encouragement was provided to ensure optimal performance.

### 2.4. Statistical Analysis

Statistical analyses were conducted using SPSS 27.0 (IBM Corp., Armonk, NY, USA). The normality of the data was assessed using the Shapiro–Wilk test. Normally distributed variables were expressed as mean ± standard deviation (SD), whereas non-normally distributed variables were presented as median and interquartile range (IQR). Categorical variables were summarized as frequencies and percentages. The relationships between muscle thickness, thickening ratio, MIP, and MEP were analyzed using Pearson’s correlation test for normally distributed data and Spearman’s correlation test for non-normally distributed data. Multiple linear regression analysis was performed to determine potential predictors of respiratory muscle strength, with *p* < 0.05 considered statistically significant.

## 3. Results

The comparison of the groups according to demographic and MIP/MEP variables is shown in Table 1. A total of 36 patients and 30 healthy individuals were included in the study. The mean age was 43.72 ± 15.75 years in the patient group and 45.66 ± 11 years in the healthy group (*p* = 0.579). No significant differences were observed in height (*p* = 0.510), weight (*p* = 0.767), or body mass index (*p* = 0.862) between the two groups. Regarding gender distribution, the patient group consisted of 24 males and 12 females, while the healthy group had 18 males and 12 females (*p* = 0.380). The prevalence of hypertension (HT) was 11.1% in the patients and 30% in the healthy individuals (*p* = 0.054), while diabetes mellitus (DM) was observed in 11.1% of the patients and 13.3% of the healthy individuals (*p* = 0.537). Coronary artery disease (CAD) was present in 5.6% of the patients and 3.3% of the healthy individuals (*p* = 0.569). Additionally, dyslipidemia was detected in 11.1% of the patients and 6.7% of the healthy individuals (*p* = 0.428). In terms of occupational distribution, 11.1% of the patients were officers, 8.3% were retired, 55.6% were workers, and 25% were housewives, while in the healthy group, 33.3% were officers, 6.7% were retired, 40% were workers, and 20% were housewives (*p* = 0.182). Smoking habits differed between groups, with 50% of the patients and 56.7% of the healthy individuals being non-smokers (*p* = 0.217). Among the smokers, 19.4% of the patients and 3.3% of the healthy individuals smoked <10 pack-years, 13.9% of the patients and 23.3% of the healthy individuals smoked 10–20 pack-years, and 16.7% in both groups smoked >20 pack-years. Additionally, alcohol consumption was reported in 22.2% of the patients and 10% of the healthy individuals (*p* = 0.160). Regarding the dominant side, the right side was dominant in 88.9% of the patients and 100% of the healthy individuals (*p* = 0.082). Maximal inspiratory pressure (MIP) was significantly lower in the patients (76.27 ± 29) than in the healthy individuals (91.63 ± 17.3, *p* = 0.007). Similarly, maximal expiratory pressure (MEP) was markedly lower in the patients (64.52 ± 21.55) compared to the healthy individuals (119.1 ± 26.48, *p* < 0.001) (Table 1).

The mean duration after injury in the patient group was 14.5 ± 12.5 months. In the analysis of the American Spinal Injury Association (ASIA) Impairment Scale (AIS) distribution, 33.3% (n = 12) of the patients were classified as AIS C, 30.6% (n = 11) as AIS A, 19.4% (n = 7) as AIS B, and 16.7% (n = 6) as AIS D. The mean functional independence measure (FIM) score of the patient group was 71.33 ± 12.57, and the mean functional ambulation scale (FAS) score was 1.25 ± 1.3.

The comparison of the right-side measurements of the groups is shown in Table 2. The right-side muscle measurements of the patient and healthy groups were compared. The M. rectus abdominis (RA) rest thickness was 9.07 ± 2.68 mm in the patients and 8.8 ± 2.04 mm in the healthy individuals (*p* = 0.733), while the RA forced thickness was 9.27 ± 2.71 mm in the patients and 9.42 ± 2.31 mm in the healthy individuals (*p* = 0.743). The RA thickening ratio was 0.07 ± 0.05 in both groups (*p* = 0.003). For the M. obliquus externus abdominis (EO), resting thickness was 5.64 ± 1.71 mm in the patients and 6.43 ± 1.94 mm in the healthy individuals (*p* = 0.122). The EO forced thickness was 5.76 ± 1.7 mm in the patients and 7.67 ± 2.35 mm in the healthy individuals, showing a statistically significant difference (*p* < 0.001). The EO thickening ratio was 0.03 ± 0.11 in the patients and 0.2 ± 0.15 in the healthy individuals (*p* < 0.001). For the M. obliquus internus abdominis (IO), resting thickness was 7.75 ± 2.07 mm in the patients and 8.44 ± 2.38 mm in the healthy individuals (*p* = 0.254). The IO forced thickness was significantly lower in the patient group (8.17 ± 2.75 mm) compared to the healthy group (11.45 ± 3.32 mm, *p* < 0.001). The IO thickening ratio was also significantly lower in the patients (0.06 ± 0.14) than in the healthy individuals (0.36 ± 0.17, *p* < 0.001). The M. transversus abdominis (TrA) rest thickness was significantly lower in the patient group (2.74 ± 0.98 mm) compared to the healthy group (3.36 ± 0.95 mm, *p* = 0.007). The TrA forced thickness was also significantly lower in the patients (3.24 ± 1.54 mm) than in the healthy individuals (5.12 ± 2.05 mm, *p* < 0.001). The TrA thickening ratio was markedly reduced in the patient group (0.14 ± 0.24) compared to the healthy group (0.63 ± 0.3, *p* < 0.001). These findings indicate that the patients exhibited significantly lower muscle thickness and thickening ratios, particularly in the EO, IO, and TrA muscles, during forced contraction compared to the healthy individuals (Table 2).

The correlation analysis of the patient group measurements with maximal inspiratory pressure (MIP) and maximal expiratory pressure (MEP) is shown in Table 3. A significant positive correlation was observed between RA end-expiratory thickness and MIP (r = 0.368, *p* = 0.027), as well as RA forced thickness and MEP (r = 0.384, *p* = 0.008). Additionally, RA forced thickness was significantly correlated with MIP (r = 0.289, *p* = 0.089). For EO, a significant positive correlation was found between EO forced thickness and MEP (r = 0.333, *p* = 0.047). However, the EO thickening ratio did not show a significant correlation with MIP (r = −0.100, *p* = 0.559) or MEP (r = −0.185, *p* = 0.281). In the IO muscle, IO forced thickness was positively correlated with MEP (r = 0.502, *p* = 0.002). A weak but significant correlation was also observed between the IO thickening ratio and MEP (r = 0.338, *p* = 0.044). For TrA, a significant correlation was found between TrA end-expiratory thickness and MEP (r = 0.508, *p* = 0.002), as well as TrA forced thickness and MEP (r = 0.530, *p* = 0.001). However, the TrA thickening ratio did not show a strong correlation with either MIP (r = 0.062, *p* = 0.720) or MEP (r = 0.428, *p* = 0.009). These findings indicate that forced contraction thickness values in RA, EO, IO, and TrA muscles are significantly associated with MIP and MEP, highlighting their potential role in respiratory muscle strength (Table 3).

The comparison of right-side muscle measurements between the T6 and above and T7–T12 levels is presented in Table 4. For RA, no significant differences were observed in RA rest thickness (*p* = 0.822) or RA forced thickness (*p* = 0.873) between the two levels. The RA thickening ratio was also similar between groups (*p* = 0.585). In EO, rest thickness (*p* = 0.875) and forced thickness (*p* = 0.876) were comparable between the T6 and above and T7-T12 levels. Similarly, the EO thickening ratio did not show significant differences (*p* = 0.797). For IO, there were no significant differences in IO rest thickness (*p* = 0.962) or IO forced thickness (*p* = 0.712). However, the IO thickening ratio was significantly higher in the T6 and above group compared to T7-T12 (*p* = 0.032). Regarding TrA, rest thickness (*p* = 0.521) and forced thickness (*p* = 0.494) were not significantly different between levels. The TrA thickening ratio was also similar (*p* = 0.451). These findings indicate that the IO thickening ratio is the only parameter that shows a significant difference between levels, with higher values in the T6 and above group. Other muscle parameters, including RA, EO, and TrA, did not show statistically significant variations between the T6 and above and T7–T12 levels (Table 4).

The comparison of USG measurements between the patients and healthy individuals is presented in Table 5. For RA, no significant differences were found between the groups in RA rest thickness (*p* = 0.733) or RA forced thickness (*p* = 0.743). However, the RA thickening ratio was significantly lower in the patient group compared to the healthy individuals (*p* = 0.003). In EO, rest thickness did not differ significantly between the groups (*p* = 0.122), but EO forced thickness was significantly lower in the patients than in the healthy individuals (*p* < 0.001). Similarly, the EO thickening ratio was significantly reduced in the patients compared to the healthy group (*p* < 0.001). For IO, rest thickness was similar between the groups (*p* = 0.254), but IO forced thickness was significantly lower in the patient group than in the healthy group (*p* < 0.001). Moreover, the IO thickening ratio was significantly lower in the patients than in the healthy individuals (*p* < 0.001). Regarding TrA, rest thickness was significantly lower in the patients compared to the healthy individuals (*p* = 0.007). Forced thickness was also lower in the patient group (*p* < 0.001). The TrA thickening ratio was significantly lower in the patients than in the healthy group (*p* < 0.001). These findings indicate that forced contraction thickness and the thickening ratios of EO, IO, and TrA muscles were significantly lower in the patients compared to the healthy individuals, suggesting weakened abdominal muscle function in the patient group (Table 5).

Intra-rater reliability of the ultrasonographic measurements was assessed by calculating the intraclass correlation coefficient (ICC) for three repeated measurements of each abdominal muscle. The ICC values demonstrated excellent consistency, ranging from 0.92 to 0.97 across all muscles. Specifically, the ICCs were 0.94 for the RA, 0.92 for the EO, 0.95 for the (IO), and 0.97 for the TrA, confirming the reproducibility of the measurements performed by a single examiner (Table 6).

## 4. Discussion

The present study investigated the predictive value of ultrasonographic abdominal muscle thickness and thickening ratios in assessing expiratory muscle strength in SCI patients. Our findings demonstrate that SCI patients exhibited significantly lower MIP and MEP values compared to healthy controls, highlighting the profound impact of spinal cord injury on respiratory muscle function. Additionally, we observed a significant reduction in the thickness and thickening ratio of the rectus abdominis (RA), external oblique (EO), internal oblique (IO), and transversus abdominis (TrA) muscles, particularly on the right side, suggesting a strong correlation between expiratory muscle weakness and abdominal muscle atrophy.

In the literature, respiratory dysfunction in SCI patients has been primarily attributed to impaired expiratory muscle function. Raab et al. reported that patients with high thoracic and cervical SCI exhibit reduced cough efficiency and increased susceptibility to pulmonary infections, which can be attributed to weakened expiratory muscles [25]. Wang et al. found that SCI patients had significantly lower peak expiratory flow (PEF) and MEP values compared to age-matched healthy individuals, which correlates with our findings of diminished respiratory muscle strength [26].

Ultrasonography has emerged as a promising tool for evaluating respiratory muscle function in various conditions. In a study by Boussuges et al., diaphragmatic thickness and thickening ratios measured by ultrasound were found to be significantly correlated with respiratory muscle strength in chronic obstructive pulmonary disease (COPD) patients [27]. Although our study focused on abdominal expiratory muscles rather than the diaphragm, the significant correlation between ultrasonographic measurements and MEP values suggests that USG can be a valuable non-invasive tool for assessing expiratory muscle dysfunction in SCI patients.

Zhu et al. evaluated the diaphragm and intercostal muscles in SCI patients using ultrasound and found a significant reduction in muscle thickness, particularly in patients with cervical SCI [28]. While their study focused primarily on inspiratory muscles, our results extend these findings by demonstrating similar ultrasound-based reductions in expiratory muscle thickness, further supporting the utility of ultrasonography in respiratory assessment.

Our results showed a strong correlation between RA, EO, IO, and TrA muscle thickness and thickening ratios with MEP values, while the correlation with MIP was weaker or absent. Wang et al. demonstrated that SCI patients with weak abdominal muscles had significantly lower MEP values, suggesting that abdominal muscle integrity is critical for effective expiratory function [29]. Wang et al. found that expiratory muscle training improved MEP values in SCI patients, further supporting the relationship between abdominal muscle integrity and respiratory strength [30].

In our study, TrA and IO muscles exhibited the most significant reductions in thickness and thickening ratios, indicating that these muscles might play a crucial role in expiratory function loss in SCI patients. Saeverud et al. and Fayssoil et al. reported that TrA activation is essential for generating expiratory pressure and maintaining core stability [31,32]. Given the pronounced weakness in TrA and IO muscles observed in our study, these muscle groups should be prioritized in targeted respiratory rehabilitation protocols to improve expiratory function and reduce pulmonary complications in SCI patients.

Our findings suggested that ultrasonographic evaluation of abdominal muscle thickness can be a useful tool for assessing respiratory dysfunction in SCI patients. Given its non-invasive, cost-effective, and widely available nature, ultrasonography may provide an alternative to spirometry and EMG-based evaluations, which require specialized equipment and greater patient cooperation. Moreover, early detection of expiratory muscle weakness through USG may allow timely implementation of respiratory rehabilitation strategies, reducing the risk of pulmonary complications.

Respiratory muscle training (RMT) has been shown to improve MIP and MEP in SCI patients. Kang et al. demonstrated that expiratory muscle training led to a significant increase in cough efficiency and peak expiratory flow, reducing the risk of respiratory infections [33]. Given our findings, future studies should explore the effectiveness of RMT in improving ultrasonographically measured abdominal muscle parameters and their impact on long-term respiratory outcomes.

### Limitations of the Study

Despite the strengths of this study, some limitations should be considered. First, the sample size was relatively small, and larger multicenter studies are needed to validate our findings. Second, ultrasonographic measurements were performed only on the dominant side, which may not fully reflect bilateral or asymmetrical muscle involvement. Future studies should incorporate bilateral ultrasonographic evaluations and examine longitudinal changes in abdominal muscle thickness with rehabilitation interventions. Additionally, combining USG with electromyographic (EMG) assessments may provide a more comprehensive understanding of expiratory muscle function in SCI patients.

## 5. Conclusions

In conclusion, our study demonstrates that SCI patients exhibit significant reductions in abdominal muscle thickness and thickening ratios, which correlate with diminished expiratory muscle strength. Ultrasonographic assessment of RA, EO, IO, and TrA muscles may serve as a valuable non-invasive tool for evaluating respiratory dysfunction in this population. These findings highlight the need for early respiratory intervention and targeted rehabilitation strategies to improve respiratory outcomes in SCI patients. Future research should explore the impact of respiratory muscle training on ultrasonographically measured muscle parameters and its clinical implications in pulmonary rehabilitation.

## Figures and Tables

**Table 1:** Comparison of groups according to demographic and MIP/MEP variables.

Variables	Patient (n = 36)	Healthy (n = 30)	*p* Values
	Mean ± SD, n (%)		
Age (Year)	43.72 ± 15.75	45.66 ± 11	0.579
Height (Cm)	170.44 ± 6.65	168.9 ± 10.18	0.510
Weight (Kg)	77.38 ± 14.3	76.96 ± 15.71	0.767
Body Mass index (BMI)	26.65 ± 4.94	26.75 ± 3.84	0.862
Gender (M/F)	24/12	18/12	0.380
Hypertension (HT)	4 (11.1%)	9 (30%)	0.054
Diabetes Mellitus (DM)	4 (11.1%)	4 (13.3%)	0.537
Coronary Artery Disease (CAD)	2 (5.6%)	1 (3.3%)	0.569
Dyslipidemia	4 (11.1%)	2 (6.7%)	0.428
Occupation			0.182
Officer	4 (11.1%)	10 (33.3%)	
Retired	3 (8.3%)	2 (6.7%)	
Worker	20 (55.6%)	12 (40%)	
Housewife	9 (25%)	6 (20%)	
Smoking			0.217
None	18 (50%)	17 (56.7%)	
<10 pack-years	7 (19.4%)	1 (3.3%)	
10–20 pack-years	5 (13.9%)	7 (23.3%)	
>20 pack-years	6 (16.7%)	5 (16.7%)	
Alcohol Consumption	8 (22.2%)	3 (10%)	0.160
Dominant Side (Right)	32 (88.9%)	30 (100%)	0.082
MIP	76.27 ± 29	91.63 ± 17.3	0.007
MEP	64.52 ± 21.55	119.1 ± 26.48	<0.001

MIP: Maximal inspiratory pressure; MEP: maximal expiratory pressure.

**Table 2:** Comparison of right-side measurements of groups.

Variables	Patient (n = 36)	Healthy (n = 30)	*p* Values
	Mean ± SD		
RA Rest	9.07 ± 2.68	8.8 ± 2.04	0.733
RA Forced	9.27 ± 2.71	9.42 ± 2.31	0.743
RA Thickening Ratio	0.07 ± 0.05	0.07 ± 0.05	0.003
EO Rest	5.64 ± 1.71	6.43 ± 1.94	0.122
EO Forced	5.76 ± 1.7	7.67 ± 2.35	<0.001
EO Thickening Ratio	0.03 ± 0.11	0.2 ± 0.15	<0.001
IO Rest	7.75 ± 2.07	8.44 ± 2.38	0.254
IO Forced	8.17 ± 2.75	11.45 ± 3.32	<0.001
IO Thickening Ratio	0.06 ± 0.14	0.36 ± 0.17	<0.001
TrA Rest	2.74 ± 0.98	3.36 ± 0.95	0.007
TrA Forced	3.24 ± 1.54	5.12 ± 2.05	<0.001
TrA Thickening Ratio	0.14 ± 0.24	0.63 ± 0.3	<0.001
RA Rest	9.07 ± 2.68	8.8 ± 2.04	0.733
RA Forced	9.27 ± 2.71	9.42 ± 2.31	0.743
RA Thickening Ratio	0.07 ± 0.05	0.07 ± 0.05	0.003

RA: M. rectus abdominis; EO: M. obliquus externus abdominis; IO: M. obliquus internus abdominis; TrA: M. transversus abdominis.

**Table 3:** Correlation analysis of patient group measurements with maximal inspiratory pressure (MIP) and maximal expiratory pressure (MEP).

		MIP	MEP
RA end-expiratory	r	0.368	0.432
	*p*	0.027	0.008
RA forced	r	0.287	0.384
	*p*	0.089	0.021
RA thickening ratio	r	−0.276	0.065
	*p*	0.103	0.707
EO end-expiratory	r	0.271	0.311
	*p*	0.109	0.065
EO forced	r	0.241	0.333
	*p*	0.157	0.047
EO thickening ratio	r	−0.101	−0.185
	*p*	0.559	0.281
IO end-expiratory	r	0.321	0.467
	*p*	0.056	0.004
IO forced	r	0.251	0.501
	*p*	0.140	0.002
IO thickening ratio	r	0.045	0.338
	*p*	0.796	0.044
TrA end-expiratory	r	0.223	0.508
	*p*	0.191	0.002
TrA forced	r	0.171	0.530
	*p*	0.319	0.001
TrA thickening ratio	r	0.062	0.428
	*p*	0.720	0.009

Spearman’s rank correlation coefficient was used due to non-normal distribution of variables. RA: M. rectus abdominis; EO: M. obliquus externus abdominis; IO: M. obliquus internus abdominis; TrA: M. transversus abdominis.

**Table 4:** Comparison of right-side muscle measurements between T6 and above and T7–T12 levels.

Variables	T6 and Above	T7–T12	*p* Values
	Mean ± SD		
RA Rest	9.34 ± 2.7	8.77 ± 2.66	0.822
RA Forced	9.45 ± 2.68	9.07 ± 2.63	0.873
RA Thickening Ratio	0.07 ± 0.05	0.06 ± 0.03	0.585
EO Rest	5.71 ± 1.7	5.64 ± 1.74	0.875
EO Forced	5.76 ± 1.7	5.64 ± 1.7	0.876
EO Thickening Ratio	0.03 ± 0.11	0.02 ± 0.1	0.797
IO Rest	7.71 ± 2.07	7.75 ± 2.06	0.962
IO Forced	8.17 ± 2.75	7.9 ± 2.7	0.712
IO Thickening Ratio	0.06 ± 0.14	0.04 ± 0.12	0.032
TrA Rest	2.74 ± 0.98	3.36 ± 0.95	0.521
TrA Forced	3.24 ± 1.54	3.68 ± 1.91	0.494
TrA Thickening Ratio	0.14 ± 0.24	0.18 ± 0.28	0.451
RA Rest	9.34 ± 2.7	8.77 ± 2.66	0.822
RA Forced	9.45 ± 2.68	9.07 ± 2.63	0.873
RA Thickening Ratio	0.07 ± 0.05	0.06 ± 0.03	0.585

RA: M. rectus abdominis; EO: M. obliquus externus abdominis; IO: M. obliquus internus abdominis; TrA: M. transversus abdominis.

**Table 5:** Comparison of USG measurements between patients and healthy individuals.

Variables	Patient (n = 36)	Healthy (n = 30)	*p* Values
	Mean ± SD		
RA Rest	9.07 ± 2.68	8.8 ± 2.04	0.733
RA Forced	9.27 ± 2.71	9.42 ± 2.31	0.743
RA Thickening Ratio	0.07 ± 0.05	0.07 ± 0.05	0.003
EO Rest	5.64 ± 1.71	6.43 ± 1.94	0.122
EO Forced	5.76 ± 1.7	7.67 ± 2.35	<0.001
EO Thickening Ratio	0.03 ± 0.11	0.2 ± 0.15	<0.001
IO Rest	7.75 ± 2.07	8.44 ± 2.38	0.254
IO Forced	8.17 ± 2.75	11.45 ± 3.32	<0.001
IO Thickening Ratio	0.06 ± 0.14	0.36 ± 0.17	<0.001
TrA Rest	2.74 ± 0.98	3.36 ± 0.95	0.007
TrA Forced	3.24 ± 1.54	5.12 ± 2.05	<0.001
TrA Thickening Ratio	0.14 ± 0.24	0.63 ± 0.3	<0.001

RA: M. rectus abdominis; EO: M. obliquus externus abdominis; IO: M. obliquus internus abdominis; TrA: M. transversus abdominis.

**Table 6:** Intraclass correlation coefficient (ICC) values indicating intra-rater reliability of ultrasonographic muscle thickness measurements.

Muscle	ICC (95% CI)	Interpretation
Rectus Abdominis (RA)	0.94 (0.89–0.97)	Excellent
External Oblique (EO)	0.92 (0.86–0.96)	Excellent
Internal Oblique (IO)	0.95 (0.91–0.98)	Excellent
Transversus Abdominis (TrA)	0.97 (0.93–0.99)	Excellent

## Data Availability

Data are available upon request to the corresponding author.
